# Dr. Kentish, on Ol. Terebinth. in Burns

**Published:** 1805-11-01

**Authors:** E. Kentish

**Affiliations:** Bristol


### *To the Editors of the Medical and Physical Journal,*

GENTLEMEN,

HAVING long since been convinced of the superiority of the treatment I have recommended in injuries, arising from an excess of heat; and having produced a mass of evidence evidence supported by the experience of others, I had left the subject to produce its own effect, and had little doubt of its general adoption by the faculty; for my own part I had arrived to that certainty of prognosis, that I could have insured the life of an individual by the treatment I recommended, and his death by any other; thus the healing art ceased to be conjectural in this instance: and were the same honest confessions of failure and success, reported in the treatment of other complaints, I trust we should make nearer advances to a certainty of practice, than we have done hitherto. From some observations in your Journal for September by Dr. Cuming, I am induced to send you a case or two which have laid by me for these three years past; though I must own, from what the Doctor has said, that I fear it will be difficult to overcome his inveterate prejudice, for so I must term it, when he asserts, "I have heard, and read much about the efficacy and superior power of ol. terebinthinæ in those cases, but all that I know of it is from report, for I never could make up my mind to use it, conceiving that it was a harsh remedy, and such an one as well calculated to produce exquisite *torture;* on this ground alone have I rejected it, as it has the appearance of applying one species of fire to extract another." Should Dr. C. favour me by perusing my Essays on this subject, he will find he has in my opinion complimented me on the choice of my remedy; and could he point out to me any substance, as he terms it, nearer fire, without being such, I shall be obliged to him, as it would more immediately fulfil my intention; but, seriously, let me ask Dr. C. can medical science be advanced by a mind which resists the admission of other's facts, because it offends a *mere opinion?* What, is the labour, experience, and observation of twenty years, supported by the experience of some of the ablest surgeons in the kingdom, to be done away by the stroke of the pen, because it accords not with the prejudiced feelings of an individual, openly, strongly, and avowedly prejudiced by his own confession? But opinions formed by prejudice will yield to facts.

Should the following case produce such an effect on Dr. Cuming's mind as to induce him to make the trial, I should not be surprized to find him as strong an advocate for the practice, as he now appears an adversary; more particularly, as he says, "facts will ever have a proper influence on his mind;" and for my own part I can desire no other. To shew him the real effects of the remedy where no prejudice could take place, a child of seven months will be a proper proper subject. Dr. M. Felix, surgeon of His Majesty's ship San Josef, in the spring of 1802, favoured me with the following case.

"My infant daughter, of seven months, was scalded by having the entire contents of a large kettle of water at the boiling point poured over her, through the carelessness of a servant letting her fall and overturning it. Fortunately I was on the spot, and as soon as we could free her from her boiling clothes, vinegar and water were applied as the readiest thing we could find to give relief; but on the suggestion of my friend Mr. Dunning, whom I had instantly called (and whose professional merits are above my praise) the spt. terebinth. was immediately procured and applied, and nine drops of tinct. opii given to her. From a state of the greatest agony, within half an hour, ease and sleep followed; and when she awoke, which was in about two hours, my dear child seemed so unconscious of pain, that she laughed, played, and seemed as much diverted, as if no such accident had happened. That this case affords decided proof of the excellence of the stimulant treatment, cannot I think be controverted, when its effects are considered on an irritable infant only seven months old, with a surface of sore almost incredible; the cuticle was entirely stript off her left arm from her shoulder to near the wrist; the left part of her back, side, abdomen, groin and thigh were all scalded; her face escaped; but her left ear, side of the neck and head, were all affected; she received also a considerable contusion on the head, from the fall on the kettle. From the heat having been detained so long upon the parts by her wetted cloaths, the effects were considerable, and it was perceived the under parts would slough; we determined therefore to persist in the stimulant plan; for thirty-six hours, cloths steeped in pure spirit of turpentine, were renewed every six hours; much symptomatic fever ensued, which we apprehended arose in great measure from the blow upon the head; a spoonful of the following was given every six hours: twenty drops of tinct. opii and liquor. volat. corn. cervi in a two-ounce mixture; and as much wine-whey as we could induce her to take; the injured parts were dressed twice a day with the following ointment, ung. ceræ ℥vj. ol. terebinth. ℥j. The fever abated; her bowels were regular; and in twenty-four hours, with the secretion of pus, all the alarming symptoms ceased; *in fine*, the sloughs threw off, and the sores healed with a rapidity truly astonishing; in less than three weeks the cure cure was compleat, all to our perfect satisfaction, and beyond all our expectations. On this fortunate case I make no comment, except that I have no hesitation in saying, that under the *old method,* or by *cold water!* I should assuredly have lost my child. To show how far we pushed the stimulant plan, my little girl (only seven months old) took in four days, sixty drops of tinct. opii and the same of liquor volat. corn. cervi, and very nearly a bottle of sherry in whey. Permit me to add, from the first sight of your publication, I was convinced of its truth; as I recollected some very successful cases during the last war, by a similar treatment; and it is now my constant mode of practice; had I entertained any doubts, what I have just related would have removed them. Allow me to conclude this long letter by requesting you to accept the best thanks of an affectionate father for the safety of a beloveed child; and believe me, with great respect, your obliged and obedient servant,

"M. Felix."

The above case is not only useful, as it shews the superiority of the mode of treatment, but in another point of view is still more valuable, as it shews, under certain circumstances, the quantity of stimulus that may be given to an infant of seven months. This case may probably be objected to by Dr. Cuming, as the treatment at the first instant was not perfectly according to my plan. Should that be the case, I will therefore point out another, which, as it happened under the inspection of a very intelligent practitioner, and one who was my assistant for some years, during the period I was assertaining the truth of the practice and principles which I have set forth at large in two Essays upon the subject, will be perfectly to the point. In the third volume of this Journal, p. 296, Mr. John Bell relates the case of an infant who was scalded by a bason of hot tea; a portion of the cuticle from the arm was brought away by the cloathes in undressing her; now, no species of burn or scald, is more painful than that which deprives the true skin of its natural covering, without destroying the skin itself, thus leaving it exposed to the action of the atmosphere, (than which nothing can be more painful) or to any application which the experience or prejudice of those who are requested to give their asistance; in this instance Mr. Bell *copiously* (his own expression) bathed the parts with ol. terebinth. previously heated, by putting the phial into hot water; this treatment was not followed by the exquisite torture Dr. C. would have expected; on the contrary, Mr. Bell enquired an *hour* after and found she was fast asleep, and had been so for some time; on his inquiring after her on the following morning she was again asleep, and had passed a remarkable good night. It would appear that this application was soothing and anodyne, instead of harsh and cruel; experience warrants its use; nor need it shock our prejudice, for if we observe what is done every day by the most experienced dentists, nothing relieves the pain of an exposed nerve so soon as some essential oil, or strong spirit; one of the most elegant of these tinctures is the Eau de Cologne, which applied to the pulpy part of a nerve of a broken or decayed tooth, on a little cotton, gives instant relief, and is followed by a pleasant soothing glow. We can scarcely conceive any thing more exquisitely sensible than the pulpy part of a nerve thus exposed; yet experience in this instance has overcome prejudice, as I make no doubt it will in the other. Investigation has been much promoted by the means of your Journal, and I hope your readers, who have it in their power, will give their assistance to determine a point of such importance.

Should Dr. C. make the quantum of pain the criterion of judging of the practice, I can have no objection; the duration of the painful stage is much prolonged by the application of cold, from all that I can learn by the experience of others; in a future communication I will illustrate this position by a strong case in point.

In the ophthalmia from cold easterly winds I can readily conceive Dr. C's aqua frigida to be an excellent application. In reading my Essay he will observe, that if I recommend hot spirit of turpentine in burns, yet there are occasions where I should recommend aqua frigida, snow, or ice. Dr. C's theory of cold might warrant some observations, but I wish to avoid dispute; I have already produced some facts in regard to the effects of heat and cold on the human body, and I have an intention of further adding to them on a future occasion.

I am, &c.

E. KENTISH, M. D.

*Bristol, Oct. 5, 1805.*

